# The effects of vitamin D supplementation on metabolic profiles and gene expression of insulin and lipid metabolism in infertile polycystic ovary syndrome candidates for in vitro fertilization

**DOI:** 10.1186/s12958-018-0413-3

**Published:** 2018-10-04

**Authors:** Majid Dastorani, Esmat Aghadavod, Naghmeh Mirhosseini, Fatemeh Foroozanfard, Shahrzad Zadeh Modarres, Mehrnush Amiri Siavashani, Zatollah Asemi

**Affiliations:** 1 Research Center for Biochemistry and Nutrition in Metabolic Diseases, Kashan University of Medical Sciences, Kashan, I.R Iran; 2 School of Public Health, University of Saskatchewan, Saskatoon, SK Canada; 3 Department of Gynecology and Obstetrics, School of Medicine, Kashan University of Medical Sciences, Kashan, I.R Iran; 4 Laser Application in Medical Science Research Center, Shahid Beheshti University of Medical Sciences, Tehran, Iran; 5 Taleghani Educational Hospital, IVF Center, Shahid Beheshti University of Medical Sciences, Tehran, Iran

**Keywords:** Vitamin D supplementation, Glycemic control, Cardio-metabolic, In vitro fertilization

## Abstract

**Background:**

Vitamin D deficiency in women diagnosed with polycystic ovary syndrome (PCOS) remarkably decreases the chance of pregnancy, which might be related to its impact on metabolic abnormalities in these patients. It is hypothesized that vitamin D supplementation influences metabolic profile of these patients and indirectly might affect fertility and the outcomes. Therefore, this study was conducted to determine the effects of vitamin D supplementation on the levels of anti-Müllerian hormone (AMH), metabolic profiles, and gene expression of insulin and lipid metabolism in infertile women with PCOS who were candidate for in vitro fertilization (IVF).

**Methods:**

This study was a randomized, double-blinded, placebo-controlled trial conducted among 40 infertile women, aged 18–40 years, diagnosed with PCOS and was candidate for IVF. Participants were randomly assigned into two intervention groups for receiving either 50,000 IU vitamin D or placebo (*n* = 20 each group) every other week for 8 weeks. Gene expression for insulin and lipid metabolism was conducted using peripheral blood mononuclear cells (PBMCs) of women with PCOS, via RT-PCR method.

**Results:**

Vitamin D supplementation led to a significant reduction in serum AMH (− 0.7 ± 1.2 vs. − 0.1 ± 0.5 ng/mL, *P* = 0.02), insulin levels (− 1.4 ± 1.6 vs. -0.3 ± 0.9 μIU/mL, *P* = 0.007), homeostatic model of assessment for insulin resistance (− 0.3 ± 0.3 vs. -0.1 ± 0.2, *P* = 0.008), and a significant increase in quantitative insulin sensitivity check index (+ 0.009 ± 0.01 vs. + 0.001 ± 0.004, *P* = 0.04), compared with the placebo. Moreover, following vitamin D supplementation there was a significant decrease in serum total- (− 5.1 ± 12.6 vs. + 2.9 ± 10.9 mg/dL, *P* = 0.03) and LDL-cholesterol levels (− 4.5 ± 10.3 vs. + 2.5 ± 10.6 mg/dL, P = 0.04) compared with the placebo.

**Conclusion:**

Overall, the findings of this trial supported that 50,000 IU vitamin D supplementation every other week for 8 weeks had beneficial effects on insulin metabolism, and lipid profile of infertile women with PCOS who are candidate for IVF. These benefits might not be evident upon having sufficient vitamin D levels.

**Trial registration:**

This study was retrospectively registered in the Iranian website (www.irct.ir) for clinical trials registration (http://www.irct.ir: IRCT20170513033941N27).

## Background

Polycystic ovary syndrome (PCOS) is one of the most common endocrine disorders among women affecting 4% to 12% of women in reproductive age [1]. It is a multifactorial syndrome presented with obesity, insulin resistance, dyslipidemia and other metabolic abnormalities. Obesity leads to more than 70% insulin resistance in these patients [2]. Insulin resistance, occurs in patients with PCOS, is significantly associated with different metabolic abnormalities including elevated aromatase activity, increased androgen production, and impaired progesterone synthesis in granulosa cells [2]. Current evidence has reported a high risk of metabolic disorders predisposing individuals to cardiovascular disease (CVD) in in vitro fertilization (IVF) pregnancies [3, 4] and animal models [5, 6]. However, the independent role of insulin resistance in in vitro fertilization (IVF) outcomes is less defined in the literature [7].

There is a significant association between serum vitamin D levels and reproductive function, already reported in murine models [8]. Further, studies investigating the role of vitamin D receptor (VDR) in reproductive tissues have supported a positive correlation between vitamin D status and reproduction [9]. VDR is located in different reproductive tissues including ovary (particularly granulosa cells), uterus, placenta and testis [10]. Diverse expression of VDR suggests a potential role of vitamin D in female reproductive function [11]. Moreover, in PCOS, ovarian physiology is influenced by the metabolic disorders observed in these patients; therefore, a beneficial effect of vitamin D on metabolic abnormalities might translate into an ideal ovarian physiology [12, 13]. Vitamin D supplementation is recommended as a potential therapeutic adjunct for the ovulatory dysfunction and metabolic disorders observed in women with PCOS [14, 15]. Evidence evaluating the impact of vitamin D deficiency on the success rate of reproduction following IVF in humans is limited [16, 17]. Moreover, vitamin D deficiency is correlated with poor ovarian stimulation in PCOS but not unexplained infertility [18]. Likewise, vitamin D has been shown to be involved in a minor pathway related to metabolic and hormonal dysregulation in women with PCOS [19]. Therefore, the beneficial effects of vitamin D on the metabolic disorders in PCOS might translate into an improvement in ovarian physiology. The majority of current studies investigating the effects of vitamin D supplementation on metabolic status in patients with PCOS have not specifically looked into IVF. And to our best knowledge, this trial is the first study assessing the effects of vitamin D supplementation on glycemic control, markers of cardiometabolic risk and gene expression of insulin and lipid metabolism in infertile women diagnosed with PCOS who were candidate for IVF. In a study conducted by Seyyed Abootorabi et al. [20] vitamin D supplementation at a dosage of 50,000 IU/week for 8 weeks reduced fasting glucose and increased adiponectin levels in women diagnosed with PCOS who were vitamin D deficient. Further, in a meta-analysis performed by Xue et al. [21], vitamin D administration to patients with PCOS significantly decreased triglycerides levels, while did not change insulin metabolism and other markers of lipid profiles. On the other hand, recently there are concerns about the risks associated to uncontrolled vitamin D supplementation [22].

Current evidence and the physiological role of vitamin D in reproductive activity might support the significance of vitamin D supplementation while doing IVF in infertile women with PCOS F. So, this study was aimed to determine the effects of vitamin D supplementation on glycemic control, markers of cardiometabolic abnormalities and gene expression of insulin and lipid metabolism in infertile women diagnosed with PCOS and candidate for IVF.

## Methods

### Trial design and participants’ characteristics

This study was a randomized, double-blinded, placebo-controlled trial conducted on 40 infertile women, aged 18 to 40 years old, with PCOS, diagnosed using Rotterdam criteria [23], who were candidate for IVF. The study was registered in the Iranian website for clinical trials registration (http://www.irct.ir: IRCT20170513033941N27). Recruited participants were referred patients at Research and Clinical Center for Infertility and Naghavi Clinic, Kashan, Iran, from December 2017 through March 2018. Any case of metabolic disorders, including thyroid disorder, diabetes or impaired glucose tolerance was excluded at screening phase. This study was performed following Declaration of Helsinki and was approved by the research ethics committee of Kashan University of Medical Sciences (KAUMS). Written informed consent was signed by all subjects prior to the intervention.

### Intervention

Participants were randomly assigned into two intervention groups to receive either 50,000 IU vitamin D (*n* = 20) or placebo (n = 20) every other week for 8 weeks. Vitamin D and placebo (paraffin) capsules were similarly matched in color, shape, size and packaging, and were manufactured by Zahravi (Tabriz, Iran) and Barij Essence (Kashan, Iran), respectively. Random number table was used for randomization by one of the researchers not involved in other processes of trial. Randomization and allocation were concealed from the investigators and study participants until the completion of analyses. Compliance rate was assessed through measuring serum 25(OH)D levels and asking patients to return back the medication containers. To increase the compliance, all subjects were received a short message on their cell phones every day, throughout the trial, to take the supplements.

### Assessment of outcomes

Insulin metabolism was considered as the primary outcome. Lipid profiles and gene expression of insulin and lipid metabolism were defined as the secondary outcomes. Fifteen milliliter fasting blood was collected at the beginning and end of the trial at Naghavi Clinic laboratory, Kashan, Iran. Fasting plasma glucose (FPG) concentrations were measured on the day of blood collection, using enzymatic kits with the coefficient variances (CVs) of less than 5%. Serum 25-hydroxyvitamin D (25 (OH) D) concentrations were determined by an ELISA kit (IDS, Boldon, UK) with inter- and intra-assay CVs of lower than 7%. Serum AMH levels were measured using an ELISA kit (Bioactiva, Homburg, Germany) with CVs of lower than 6%. Serum insulin concentrations were measured by an ELISA kit (DiaMetra, Milano, Italy) with inter- and intra-assay CVs of 3.5 and 5.0%, respectively. The homeostasis model of assessment-insulin resistance (HOMA-IR) and the quantitative insulin sensitivity check index (QUICKI) were determined according to the published formula [24]. Enzymatic kits (Pars Azmun, Tehran, Iran) were applied to measure serum lipid profiles with inter- and intra-assay of less than 5%.

### Isolation of lymphocyte cells

Blood samples were used to extract lymphocyte cells, using 50% percoll (Sigma-Aldrich, Dorset, UK). Cell count and viability test were performed using trypan blue, RNA and DNA extraction [25].

### RNA extraction and real-time PCR (RT-PCR)

RNA was extracted from blood samples using RNX-plus kit (Cinnacolon, Tehran, Iran). RNA suspension was frozen at − 20 °C until cDNA was derived. RNA quantification was conducted using UV spectrophotometer and total RNAs were extracted from each sample. Each sample OD 260/280 ratio was considered to range from 1.7 to 2.1, representing no contamination with either protein or DNA [25].

The isolated RNA was reverse transcribed to cDNA library, using moloney murine leukemia virus reverse transcriptase (RT). Gene expressions of peroxisome proliferator-activated receptor gamma (PPAR-γ), glucose transporter 1 (GLUT-1) and low-density lipoprotein receptor (LDLR) were done using peripheral blood mononuclear cells (PBMCs) via SYBR green detection and Amplicon Kit, applying quantitative RT-PCR and Light Cycler technology (Roche Diagnostics, Rotkreuz, Switzerland) **(**Table 1)**.** Glyceraldehyde-3-phosphate dehydrogenase (GAPDH) primers were used as a housekeeping gene. Primers were designed using Primer Express Software (Applied Biosystems, Foster City, USA) and Beacon designer software (Takaposizt, Tehran, Iran). Relative transcription levels were calculated using Pffafi or 2^-∆∆CT^ methods [25].

**Table 1:** Specific primers used for real-time quantitative PCR

Gene	Primer	Product size (bp)	Annealing temperature ©
GAPDH	F: AAGCTCATTTCCTGGTATGACAACG	126	61.3
	R: TCTTCCTCTTGTGCTCTTGCTGG		
PPAR-γ	F: ATGACAGACCTCAGACAGATTG	210	54
	R: AATGTTGGCAGTGGCTCAG		
GLUT-1	F: TATCTGAGCATCGTGGCCAT	238	62.1
	R: AAGACGTAGGGACCACACAG		
LDLR	F: ACTTACGGACAGACAGACAG	223	57
	R: GGCCACACATCCCATGATTC		

*GAPDH* glyceraldehyde-3-Phosphate dehydrogenase, *GLUT-1* glucose transporter 1, *LDLR* low-density lipoprotein receptor, *PPAR-γ* peroxisome proliferator-activated receptor gamma

### Sample size

Clinical trial sample size formula was used to calculate sample size considering a type one error (α) of 0.05 and type two error (β) of 0.20 with the power of 80%. Mean difference (d) of HOMA-IR equal to 0.8 and SD of 0.8 were used for the calculation [26]. Sample size was defined as 16 participants in each group. Considering 4 probable dropouts in each group, the final sample size was determined to be 20 participants in each group.

### Statistical methods

Kolmogorov-Smirnov test was used to assess the normal distribution of variables. Analyses were replicated using intention-to-treat (ITT) approach. To determine the differences in anthropometric measures and gene expression of insulin and lipid metabolism between the intervention groups, we used independent samples *t*-test. The effects of vitamin D supplementation on glycemic control and markers of cardiometabolic risk were assessed using repeated measures ANOVA test. The *P*-values of < 0.05 were considered statistically significant. Statistical analyses were conducted using the Statistical Package for Social Science version 18 (SPSS Inc., Chicago, Illinois, USA).

## Results

**Fig. 1:**
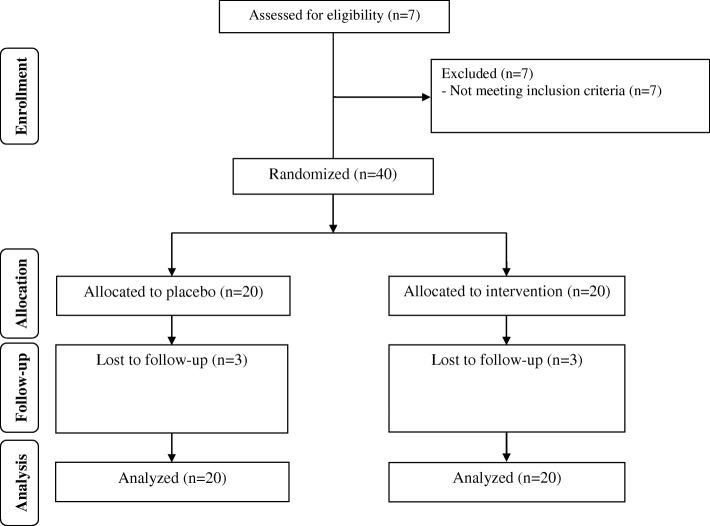
Summary of patient flow diagram

We had three dropouts in each intervention group due to personal reasons, and 34 participants [infertile women with PCOS candidate for IVF receiving vitamin D (*n* = 17) and placebo (n = 17)] completed the study (Fig. 1). Using ITT protocol, all 40 participants (20 in each group) were included in the final analysis. Compliance rate in this trial ranged 90% to100% in both groups. No side effects were reported following vitamin D supplementation in infertile women with PCOS who were candidate for IVF throughout the study. Mean age, height, weight and BMI were not statistically different between the intervention groups throughout the trial (Table 2).

**Table 2:** General characteristics of study participants

	Placebo group (n = 20)	Vitamin D group (n = 20)	P^a^
Age (y)	30.1 ± 3.4	29.9 ± 4.4	0.87
Height (cm)	159.2 ± 2.0	160.4 ± 3.3	0.16
Weight at study baseline (kg)	72.0 ± 6.9	71.3 ± 9.9	0.77
Weight at end-of-trial (kg)	72.2 ± 6.8	71.5 ± 9.6	0.79
Weight change (kg)	0.1 ± 0.8	0.2 ± 0.9	0.70
BMI at study baseline (kg/m^2^)	28.4 ± 2.6	27.7 ± 3.9	0.49
BMI at end-of-trial (kg/m^2^)	28.5 ± 2.6	27.8 ± 3.7	0.50
BMI change (kg/m^2^)	0.1 ± 0.3	0.1 ± 0.3	0.68

Data are means± SDs

^a^ Obtained from independent *t*-test

After 8-week intervention, vitamin D supplementation led to a significant reduction in serum AMH (− 0.7 ± 1.2 vs. -0.1 ± 0.5 ng/mL, *P* = 0.02) and insulin levels (− 1.4 ± 1.6 vs. -0.3 ± 0.9 μIU/mL, *P* = 0.007), HOMA-IR (− 0.3 ± 0.3 vs. -0.1 ± 0.2, *P* = 0.008), as well as a significant increase in QUICKI (+ 0.009 ± 0.01 vs. + 0.001 ± 0.004, *P* = 0.04) compared with placebo (Table 3). Further, taking vitamin D supplements significantly decreased serum total- (− 5.1 ± 12.6 vs. + 2.9 ± 10.9 mg/dL, *P* = 0.03) and LDL-cholesterol levels (− 4.5 ± 10.3 vs. + 2.5 ± 10.6 mg/dL, P = 0.04) compared with the placebo. There was no significant effect of vitamin D supplementation on fasting glucose and other parameters of lipid profiles.

**Table 3:** Glycemic control, markers of cardio-metabolic risk and oxidative stress at baseline and after the 8-week intervention in infertile polycystic ovary syndrome women candidate for in vitro fertilization that received either vitamin D supplements or placebo

	Placebo group (n = 20)			Vitamin D group (*n* = 20)			P^a^
	Baseline	End-of-trial	Change	Baseline	End-of-trial	Change	
Vitamin D (ng/mL)	11.0 ± 2.4	10.9 ± 2.1	-0.1 ± 0.6	10.5 ± 2.5	21.7 ± 5.9	11.2 ± 5.0	< 0.001
AMH (ng/mL)	8.7 ± 2.7	8.6 ± 2.5	-0.1 ± 0.5	7.7 ± 3.4	7.0 ± 3.1	−0.7 ± 1.2	0.02
FPG (mg/dL)	92.9 ± 5.5	93.5 ± 5.6	0.5 ± 3.0	90.3 ± 10.5	89.4 ± 10.6	−0.9 ± 7.4	0.42
Insulin (μIU/mL)	11.4 ± 1.9	11.1 ± 2.0	−0.3 ± 0.9	11.2 ± 2.2	9.8 ± 2.7	−1.4 ± 1.6	0.007
HOMA-IR	2.6 ± 0.5	2.5 ± 0.4	−0.1 ± 0.2	2.5 ± 0.7	2.2 ± 0.7	−0.3 ± 0.3	0.008
QUICKI	0.33 ± 0.008	0.33 ± 0.009	0.001 ± 0.004	0.33 ± 0.01	0.34 ± 0.02	0.009 ± 0.01	0.04
Triglycerides (mg/dL)	111.5 ± 35.5	117.4 ± 34.8	5.9 ± 13.2	105.3 ± 33.5	107.5 ± 38.1	2.1 ± 17.4	0.44
VLDL-cholesterol (mg/dL)	22.3 ± 7.1	23.5 ± 6.9	1.2 ± 2.6	21.1 ± 6.7	21.5 ± 7.6	0.4 ± 3.5	0.44
Total cholesterol (mg/dL)	197.1 ± 36.3	200.0 ± 36.5	2.9 ± 10.9	203.6 ± 26.6	198.5 ± 24.7	−5.1 ± 12.6	0.03
LDL-cholesterol (mg/dL)	124.9 ± 35.9	127.4 ± 35.3	2.5 ± 10.6	133.1 ± 21.1	128.7 ± 20.8	−4.5 ± 10.3	0.04
HDL-cholesterol (mg/dL)	49.9 ± 7.5	49.1 ± 8.2	−0.8 ± 3.9	49.4 ± 5.7	48.4 ± 5.4	−1.0 ± 2.8	0.81

Data are means± SDs

^a^ Obtained from repeated measures ANOVA test

*AMH* anti-Müllerian hormone, *FPG* fasting plasma glucose, *HOMA-IR* homeostasis model of assessment-estimated insulin resistance, *QUICKI* quantitative insulin sensitivity check index

**Fig. 2:**
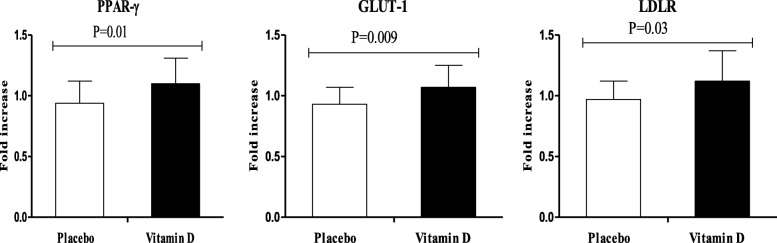
Effect of 8-week supplementation with vitamin D or placebo on expression ratio of PPAR-γ, GLUT-1 and LDLR gene in PBMCs of infertile women with polycystic ovary syndrome who were candidate for in vitro fertilization**.** GLUT-1, glucose transporter 1; LDLR, low-density lipoprotein receptor; PBMCs, peripheral blood mononuclear cells; PPAR-γ, peroxisome proliferator-activated receptor gamma

RT-PCR quantitative tests showed a significant upregulation of gene expression of PPAR-γ (*P* = 0.01), GLUT-1 (*P* = 0.009) and LDLR (P = 0.03) in PBMCs of infertile women diagnosed with PCOS who were candidate for IVF following vitamin D supplementation rather than placebo (Fig. 2).

## Discussion

The results of this trial demonstrated the beneficial effects of 50,000 IU vitamin D supplementation every other week for 8 weeks on improving insulin metabolism and some of the markers of lipid profile among infertile women diagnosed with PCOS who were candidate for IVF. These benefits might not be evident upon having sufficient vitamin D levels. To our best knowledge, this study has reported the effects of vitamin D supplementation on AMH, glycemic control, lipid profiles and gene expression of insulin and lipid metabolism in infertile women for the first time.

Polycystic ovary syndrome predisposes patients to different metabolic abnormalities, such as insulin resistance and dyslipidemia [27, 28]. This study demonstrated that 50,000 IU vitamin D supplementation every other week for 8 weeks resulted in significant decreases in serum AMH, insulin levels and HOMA-IR score, and a significant increase in QUICKI in infertile women with PCOS who were candidate for IVF. Further, vitamin D supplementation significantly increased gene expression levels of PPAR-γ and GLUT-1. In the agreement with our study, Gupta et al. [29] demonstrated that vitamin D supplementation at a dosage of 60,000 IU weekly for 12 weeks significantly reduced insulin levels and HOMA-IR, and significantly increased QUICKI in vitamin D-deficient women with PCOS. In another study, taking vitamin D supplements at a dosage of 20,000 IU per week for 24 weeks by women with PCOS, having serum calcium levels < 2.65 mmol/L and uncertain serum 25 (OH) D status at baseline, improved glucose metabolism and menstrual frequency [30]. However, there are also studies with discrepant findings. Garg et al. [31], showed that vitamin D supplementation at a dose of 4000 IU/day for 6 months to vitamin D-deficient women with PCOS did not have any significant effect on insulin resistance and insulin secretion. Insulin resistance is associated with increased synthesis of vasoconstriction factors, which might be the reason for vascular stiffness in women diagnosed with PCOS [32]. Therefore, improved insulin metabolism by vitamin D supplementation may decrease the risk of metabolic complications subsequent to insulin resistance in these patients. The mechanisms involved in the impact of vitamin D on insulin metabolism could be direct and indirect effects of vitamin D on stimulating insulin release through improved vitamin D receptor expression, enhancing insulin sensitivity for glucose transportation and suppressing the release of pro-inflammatory cytokines [33].

We found that 50,000 IU vitamin D supplementation every other week for 8 weeks significantly decreased total- and LDL-cholesterol levels in infertile women with PCOS who were candidate for IVF, compared with the placebo, though other lipid profiles parameters remained unchanged. Moreover, vitamin D supplementation significantly increased gene expression levels of LDLR. Similar to our findings, vitamin D supplementation at a dosage of 4000 IU/day for 12 weeks significantly decreased total cholesterol concentrations in vitamin D deficient HIV-infected patients [34]. In a meta-analysis conducted by Akbari et al. [35], vitamin D supplementation significantly decreased LDL-cholesterol levels in gestational diabetes patients, though other lipid profiles parameters did not change. There are also contrary studies demonstrating that vitamin D supplementation (100,000 IU loading dose, followed by 20,000 IU/week) did not improve CVD risk factors including blood pressure, lipid profiles and glucose metabolism parameters in vitamin D insufficient population [36]. Current evidence suggested that increased insulin and androgens levels may negatively influence lipid profiles in women with PCOS [37]. Fatty acids byproducts have an important role in inflammation and reproduction [37]. Improved insulin sensitivity and decreased production of PTH by vitamin D might decrease total- and LDL-cholesterol [38]. Further, insulin reduces biosynthesis of cholesterol via increased 3-hydroxy-3-methylglutaryl CoA (HMG-CoA) reductase activity [39], which in turn decreases total- and LDL-cholesterol levels.

There are a few limitations in this study. First, the evaluation of insulin resistance in the current study, was only based on HOMA-IR. We did not assess any direct dynamic test, such as glucose tolerance test or hyperinsulinemic euglycemic clamp. Second, some of the insignificant results might be related to the short duration of intervention. So, further studies are required with longer duration and higher sample size to confirm our findings. In addition, all the participants were vitamin D deficient, so the observed effects might be correlated with the correction of vitamin D deficiency, rather than the supplementation per se. This should be considered in the interpretation of our finding.

## Conclusions

Overall, the findings of this trial supported that 50,000 IU vitamin D supplementation every other week for 8 weeks had beneficial effects on insulin metabolism, and some parameters of lipid profiles among infertile women with PCOS who were candidate for IVF. These benefits might not be evident upon having sufficient vitamin D levels.

## Abbreviations

25 (OH) D: 25-hydroxyvitamin D
AMH: anti-Müllerian hormone
FPG: fasting plasma glucose
GAPDH: glyceraldehyde-3-phosphate dehydrogenase
HOMA-IR: homeostasis model of assessment-estimated insulin resistance
HMG-CoA: 3-hydroxy-3-methylglutaryl CoA
ITT: intention-to-treat
QUICKI: quantitative insulin sensitivity check index
GLUT-1: glucose transporter 1
LDLR: low-density lipoprotein receptor
PBMCs: peripheral blood mononuclear cells
PPAR-γ: peroxisome proliferator-activated receptor gamma
